# Heightened Distraction under Competition in Obsessive-Compulsive Disorder

**DOI:** 10.64898/2026.03.15.711932

**Published:** 2026-03-17

**Authors:** Katherine J. McCain, Estelle Ayomen, Arash Mirifar, Heather Simpson Martin, Dominic Demeterfi, Daniel J. McNeil, Gian DePamphilis, Rami Hatem, Robyn Nelson, Grace Melville, Emma Hammes, Alexandra Lee, Ryan McCarty, Mindy Le, Catherine Paciotti, Patricia Coutinho, Carol A. Mathews, Andreas Keil

**Affiliations:** 1 Department of Psychology, University of Florida, Gainesville, FL; 2 Department of Psychiatry, University of Florida, Gainesville, FL; 3 Center for OCD, Anxiety, and Related Disorders, University of Florida, Gainesville, FL; 4 Evelyn F. and William L. McKnight Brain Institute, University of Florida, Gainesville, FL; 5 Norman Fixel Institute for Neurological Diseases, University of Florida, Gainesville, FL; 6 Department of Clinical and Health Psychology, University of Florida, Gainesville, FL; 7 Department of Psychiatry, Thomas Jefferson University Hospital, Philadelphia, PA; 8 Department of Psychiatry and Behavioral Sciences, Baylor College of Medicine, Houston, TX; 9 Department of Psychiatry, University of South Florida, Tampa, FL

**Keywords:** Visuocortical competition, attention, ssVEP, obsessive-compulsive disorder

## Abstract

The identification of objective, dimensional indices of mental health is of central importance in the pursuit of transdiagnostic multi-dimensional frameworks of psychopathology. Altered visual processing occupies a specific domain of interest and motivated the present investigation aimed to quantify the visuocortical impact of affective naturalistic distractor cues on limited capacity attentional resources in obsessive-compulsive disorder (OCD). The current investigation examined the extent to which attentional resources are allocated toward task cues under affective and disorder-relevant distraction in participants with OCD (N = 33) and control participants (N = 31). Steady-state visual evoked potentials (ssVEPs) in response to task-relevant cues were examined using a foreground task where participants detected coherent motion in a flickering random dot kinematogram (RDK) overlaid on naturalistic distractor pictures ranging in emotional content (pleasant, neutral, unpleasant, and OCD-evoking pictures). Amplitude envelopes of ssVEPs in response to the motion stimulus served as an index of visuocortical engagement with task-relevant cues. Data were also fitted to the distraction under competition model (DUC), a computational framework of attention selection. Group differences emerged with stronger visuocortical competition effects (attenuated task engagement) for the OCD group, driven largely by the unpleasant pictures, followed by the OCD-evoking pictures. Furthermore, the DUC model fit well in both groups, demonstrated the dominance of the visuocortical competition observed in response to the unpleasant pictures, and revealed the presence of substantial competition in response to the OCD-evoking pictures in the OCD group.

## Introduction

Exhibiting a lifetime prevalence of approximately 2% (Fawcett et al., 2020), obsessive-compulsive disorder (OCD) is characterized by experiencing obsessive, unwanted thoughts and completing compulsions to alleviate feelings of dread, shame, or discomfort associated with the obsession (Abramowitz et al., 2009; Lack, 2012; Veale & Roberts, 2014). Both obsessions and compulsions can become time-consuming and ritualistic, which can be highly distressing for individuals, increasing the saliency of obsessional contexts and the diversion from goal-driven behavior (Eisen et al., 2006; Moritz, 2008). To this end, early visual perception is often heightened in individuals with OCD and may be indicative of hypervigilance (Chapman et al., 2023; Pallanti et al., 2009; Thomas et al., 2013; Zhang et al., 2017). In addition, evidence of altered visual attention processes has emerged across numerous investigations employing cognitive and behavioral tasks aimed to examine visual processing of neutral, emotional, and disorder-relevant stimuli in participants with OCD (for a review, see Ayomen et al., 2025). However, no investigation to date has examined the extent to which distracting visual stimuli ranging in emotional and disorder relevance compete with goal-directed (task-relevant) cues for visual attention resources in individuals with OCD and controls. The identification of robust, objective markers of altered visual attention implementation in OCD may contribute to our understanding of the neurocognitive basis of OCD and the development of visual domain specific indices of obsessive-compulsive symptoms (OC-symptoms).

The present study used steady-state visual evoked potentials (ssVEPs) to quantitatively and continuously assess competition effects between a neutral foreground task and task-irrelevant distractor pictures varying in emotional content. The current task was adapted from Müller et al. (2008) and included a task-relevant coherent motion detection stimulus (random dot kinematogram; RDK) overlaid on naturalistic distractor pictures ranging in emotional content, including disorder-relevant (OCD-evoking), pleasant, unpleasant, and neutral pictures. The motion stimulus (RDK) was flickered at a rate of 8.57 Hz and functioned to evoke oscillatory brain responses (ssVEPs) which served as an index of visuocortical engagement with task-relevant cues. Leveraging competitive interactions in visual cortex offers insight into the prioritization of visual information (Beck & Kastner, 2009; Kastner & Ungerleider, 2001) and may provide dimensional indices of the implementation of limited capacity attention resources in individuals with OCD.

The implementation of selective attention can be described as the allocation of attentional resources toward competing sensory inputs and functions to enhance selective inputs while suppressing others (Luck & Gold, 2008). Under the biased competition framework (Desimone & Duncan, 1995), competition between visual inputs is resolved by attention selection when salience alone cannot resolve competition (Beck & Kastner, 2009; Kastner & Ungerleider, 2000). For instance, attention selection can modulate visuocortical engagement across competing visual inputs at the level of visual cortex (Hillyard & Anllo-Vento, 1998; Keil et al., 2005), evoking interactions often best measured between overlapping stimuli. Relevant to the present study, characteristics of the stimulus including the emotional or motivational relevance of competing visual streams can facilitate or bias the processing of those visual inputs, often at the expense of neutral inputs (Bradley et al., 2012; Desimone & Duncan, 1995; Yiend, 2010).

Affective interstimulus competition paradigms optimize competitive interactions in visual cortex and have been used to examine the cost effect of attention biases toward affective task-irrelevant cues and the resulting competition for visuocortical representation (Bradley et al., 2012; Müller et al., 2026). A foundational example of work aimed to quantify the attentional trade-off between task and task-irrelevant distractor cues was conducted by Müller and colleagues (2008). The authors developed a distraction under competition paradigm which included a foreground change detection task overlaid on naturalistic pictures ranging in emotional valence. Visuocortical trade-off between competing cues was indexed by the ssVEP response to the task cues, where decreased ssVEP amplitude indicated an attentional bias toward the task-irrelevant pictures. The authors demonstrated that affective distractor stimuli compete for visual attention resources more so than neutral stimuli, with competition effects emerging early in the trial (estimated to be no earlier than 100 ms) and extending around one second after picture onset (Müller et al., 2008). Early affective facilitation, indexed by ssVEP envelope attenuation, was observed in similar iterations of the distraction under competition paradigm, beginning around 400 ms and persisting for about 300 ms (Hindi Attar et al., 2010) or 600 ms (Schönwald & Müller, 2014). Providing a continuous measure of visuocortical engagement, the ssVEP technique is particularly well suited to examine competitive interactions in primary visual cortex in non-clinical and clinical samples (De Echegaray et al., 2024; Deweese et al., 2014; Müller et al., 2008; Wieser et al., 2011, 2012, 2016).

Other investigations have employed similar distraction under competition tasks and have found sustained visuocortical competition in response to affective distractor pictures in clinical samples. For instance, Wieser and colleagues (2012) found evidence of increased visuocortical competition effects in participants with high social anxiety in response to angry faces, compared to pleasant and neutral faces. These competition effects were detected early into the trial (500 ms to 1000 ms) indicating the presence of initial attention capture demonstrated in Müller et al. (2008). Furthermore, following initial attention capture, a sustained period of attention selection toward the task-irrelevant angry faces persisted for another two seconds. Deweese and colleagues (2014) employed a version of the present task to examine competitive interactions between task cues and task-irrelevant distractor pictures ranging in emotional content and disorder relevance in individuals endorsing snake phobia. Participants exhibiting high snake phobia symptoms showed sustained competition in response to the snake pictures, when compared to disorder-irrelevant unpleasant pictures, extending several seconds after picture onset. Critically, in both cases, this pattern of sustained competition was specific to the presence of disorder-relevant cues with a negative emotional valence.

Investigations examining the impact of disorder-relevant cues on visual attention processes indexed by the late positive potential (LPP), a measure of sustained attention toward affective cues (Hajcak et al., 2012), have revealed conflicting findings in participants with OCD. Zhang and colleagues (2017) reported reduced LPP amplitudes in response to disorder-relevant and threat related words in participants with OCD compared to controls. In contrast, Paul and colleagues (2016) showed increased LPP amplitudes in response to disorder-relevant pictures in participants with OCD compared to controls. Most notably, Paul and colleagues (2016) revealed heightened LPP amplitude in response to aversive pictures compared to the amplitude in response to disorder-relevant pictures within OCD participants. Similarly, Dieterich and colleagues (2017) found significant enhancement of LPP amplitudes in potential threat conditions in participants with OCD compared to controls. Resembling the OCD-evoking picture themes identified by Paul et al. (2016), the present study included OCD-evoking pictures covering several OCD symptom categories. Disorder-relevant images were chosen to primarily elicit OCD symptoms in participants who may have endorsed obsessions or compulsions related to aggressive, contamination, cleaning/washing, checking, symmetry, or counting. Given that obsessions and compulsions across individuals are more idiosyncratic than fears in specific phobia and social anxiety disorder (Broekhuizen et al., 2023; Brooks et al., 2018; Moritz et al., 2009), evoking attention biases in participants with OCD warrants the inclusion of multiple symptom-provoking dimensions. Some pictures will be neutral to many observers, because they may include content that, for example, is related to symmetry, checking, or counting, not known to elicit aversive responses.

To further elucidate the visuocortical trade-off between task and task-irrelevant affective and disorder-relevant distractors, the present study quantified visuocortical competition by applying the distraction under competition model (DUC; De Echegaray et al., 2024; Müller et al., 2026), a framework for selective attention. The biased competition (Desimone & Duncan, 1995) and sensory gain amplification (Hillyard et al., 1998) conceptual models of selective attention were integrated under this framework (De Echegaray et al., 2024; Müller et al., 2026). This is accomplished by modeling visuocortical facilitation, as a result of feedforward processing amplifying initial stimulus gain of the more salient stimulus, and the subsequent competition for visuocortical representation between the task and distractor cues (De Echegaray et al., 2024; Müller et al., 2026). The DUC model functions to parametrize dynamic competitive interactions between task-evoked and distractor-evoked responses in visual cortex (De Echegaray et al., 2024; Müller et al., 2026). Therefore, for the present application of the model, picture-evoked responses were isolated from the oscillatory signal to serve as the distractor-evoked response. Modeling visuocortical competition can integrate several characteristics of competitive interactions into meaningful parameter estimates to enhance the reliability of our findings (Haines et al., 2025).

Given the salient nature of the affective naturalistic scenes (Wöstmann et al., 2022), we expected decreased visuocortical engagement or attenuation of the task-evoked ssVEP envelope following affective distractor picture onset, relative to neutral picture onset, regardless of group. Furthermore, we expected to observe group differences in the cost effect of attentional biases toward affective and disorder-relevant distractor pictures and hypothesized that these differences would be driven by specific conditions. For instance, the pleasant, unpleasant, and OCD-evoking picture conditions were expected to prompt greater decreases in task-evoked visuocortical engagement in the OCD group compared to the control group. We also anticipated poorer task performance on trials where unpleasant, pleasant, and disorder-relevant distractors were presented for the OCD group compared to the control group. Finally, we expected the DUC model to fit the empirical data, corroborating an attentional shift from task cues to affective and disorder-relevant distractors and indicating greater visuocortical competition for the OCD group compared to the control group.

## Methods

### Participants

Thirty-three participants with OCD (27.42 ± 9.35 years) and 31 control participants (38.13 ± 18.97) were recruited from psychiatry and psychology clinics at the University of Florida and through flyers placed in the local community (see Table 1 for demographic data). All activities related to this research project were completed as a part of a large-scale research initiative investigating obsessive-compulsive anxiety spectrum disorders and approved by the University of Florida International Review Board in accordance with the Declaration of Helsinki.

### Clinical assessments

Participants were assessed using a battery of self-report questionnaires and underwent a semi-structured clinical interview conducted by a clinician trained in research procedures. OCD was assessed using the Yale-Brown Obsessive Compulsive Scale (Y-BOCS; Goodman et al., 1989) the Obsessive-Compulsive Inventory (OCI-R; Foa et al. 2002), and the Mini Neuropsychiatric Interview (MINI; Sheehan et al., 1998). The Structured Interview for Hoarding Disorder (SIHD; Nordsletten et al., 2013) and Saving Inventory-revised (SI-R; Frost et al., 2004) was used to assess for hoarding disorder. The SNAP-IV (Bussing et al., 2008) was used to assess for Attention Deficit and Hyperactivity Disorder (ADHD). Medical records, when available, were obtained to provide additional clinical information. A best estimate process was used to assign DSM-5 diagnoses. Best estimators were blinded to the case/control status of the participant and reviewed all available clinical data (i.e., self-report questionnaires, clinical interviews, and medical records; Nutley et al., 2020) for a given participant. Assignments of “definite,” “probable,” “not present,” or “not determined” (e.g, not enough data were present to make a determination) were made for each DSM-5-TR diagnosis. In the event of probable diagnoses, two best estimates were independently completed, and a subsequent consensus diagnosis was reached. In the event that a consensus could not be reached between two best estimators, a third best estimator was assigned.

### Inclusion and exclusion criteria

Participants were included in the study if they were 18 years of age or older. OCD participants were required to meet criteria for a lifetime history of OCD as defined by the DSM-5. Control participants were included if they did not meet criteria for OCD. Those with psychosis, known intellectual disability, head trauma with loss of consciousness, or medical or neurological conditions known or suspected to affect cognitive function were also excluded. A lifetime or current history of other psychiatric diagnoses were not exclusionary in either group. Participants with fewer than 50% of trials remaining in any condition after EEG artifact rejection, were excluded from the analysis. Finally, participants exhibiting less than 50% task accuracy across all picture conditions were excluded from the analysis.

### Stimuli and procedure

**Figure 1.**
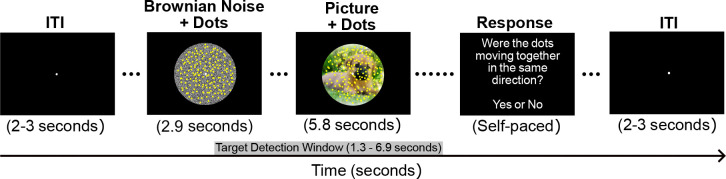
Distraction under competition task. Trials began with a period of Brownian noise (2.90 s). The distraction under competition period (5.80 s) was marked by the onset of the distractor pictures subtending the same annulus defined in the Brownian noise period combined with the continuation of the motion stimulus overlayed. On target trials half of the dots moved together in coherent motion in one direction (45°, 135°, 225°, or 315°) once per trial (target detection window). On non-target trials, the dots never moved in the same direction. Following each trial, participants were asked to indicate whether they detected coherent motion using the mouse to select ‘yes’ or ‘no’ during the self-paced response period.

Visual stimuli were created in Psychtoolbox for Matlab (version 3.0.18) and presented on a Samsung LS23A950 Monitor with a 120 Hz refresh rate and resolution of 1920 × 1080. The distraction under competition paradigm included a foreground coherent motion detection task overlaid on naturalistic distractor pictures ranging in emotional content and disorder relevance. The motion stimulus used in the foreground task was created using a random dot kinematogram algorithm that dictated the original position of the moving stimuli, the frequency of the random movement, and the frequency of the coherent movement. The motion stimulus included 150 bright yellow dots (0.29° of visual angle; see Figure 1) constrained by a central circular region of the screen (9.20° of visual angle) and maintained constant motion while flickering at a frequency of 8.57 Hz. Pleasant, neutral, and unpleasant distractor pictures were chosen from the International Affective Picture Set (IAPS; Lang et al. 1997), and OCD-evoking pictures were carefully curated from a database of openly licensed pictures. OCD-evoking pictures were chosen to primarily elicit OCD symptoms in participants who endorsed obsessions or compulsions related to aggressive, contamination, cleaning/washing, checking, symmetry, or counting (Goodman et al., 1989).

**Figure 2.**
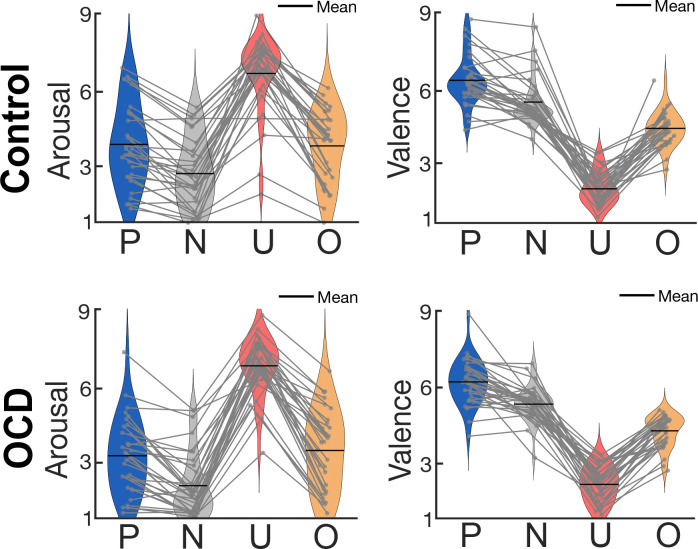
Subjective valence and arousal ratings across conditions (pleasant, P; neutral, N; unpleasant, U; OCD-evoking, O) using the self-assessment manikin (SAM). For valence, a rating of 1 indicated negative valence (unpleasant) and a rating of 9 indicated positive valence (pleasant). For arousal, a rating of 1 indicated low arousal (evoking a feeling of calm) and a rating of 9 indicated high arousal (evoking excitement). Light gray dots and the lines connecting them indicated individual subject average arousal and valence ratings for each condition.

Trials began with a period of Brownian noise (2.90 s) where the motion stimulus was superimposed over a central circular background with the RBG value of each pixel ranging randomly between 0–255, which created the perception of a gray-scale background (9.20° of visual angle; see Figure 1). The Brownian noise period functioned to attenuate transient visuocortical responses to luminance changes at the onset of the distractor pictures behind the motion stimulus (Deweese et al., 2014). The distraction under competition period (5.80 s) was marked by the onset of the distractor pictures subtending the same annulus defined in the Brownian noise period combined with the continuation of the motion stimulus overlaid (see Figure 1). Throughout the trial, the motion stimulus was presented for a total of 8.70 seconds, and participants were asked to maintain fixation by directing their gaze to a white fixation dot in the center of the screen (0.34° of visual angle; see Figure 1). On target trials, half of the dots could move together in coherent motion in one direction (45°, 135°, 225°, or 315°) once per trial, and this coherent motion could only occur between 1.3 and 6.9 seconds (coherent motion detection time window) into the trial. On non-target trials, the dots never moved in the same direction, maintaining constant random motion throughout. The task consisted of 30 trials in each of the four picture conditions. Target and non-target trials were evenly distributed so all picture conditions included 15 target and 15 non-target trials that were then randomized within the conditions. Following each trial, participants were asked to indicate whether they detected coherent motion using the mouse to select ‘yes’ or ‘no’ during the self-paced response period. After the self-paced response period, the inter-trial interval ranged from 2–3 seconds. The task began with a brief practice, and upon completion of the task, participants provided subjective valence and arousal ratings for each picture (see Figure 2) using the 9-point Self-Assessment Manikin scale (SAM; Bradley & Lang, 1994). For valence, a rating of 1 indicated negative valence (unpleasant) and a rating of 9 indicated positive valence (pleasant). For arousal, a rating of 1 indicated low arousal (evoking a feeling of calm) and a rating of 9 indicated high arousal (evoking excitement).

### EEG data acquisition

Participants were seated in an electrically shielded recording chamber 100 cm from the stimulus presentation monitor. Continuous EEG data was recorded using an electrical geodesics (EGI) high-density, high impedance 257 channel system. Online recording parameters included sensor Cz as the online reference and a sampling rate of 1000 Hz. In addition, impedance levels were kept below 60 kΩ throughout the recording.

### EEG data processing

Offline, the continuous EEG data were processed using custom automated preprocessing and postprocessing pipelines created using the EEGLAB MATLAB toolbox (Delorme & Makeig, 2004) and are openly available in the project repository (https://osf.io/s73d5/overview).

#### Task-evoked responses

##### Preprocessing.

The preprocessing pipeline began by down sampling the continuous EEG data from 1000 Hz to 500 Hz. Next, a 3 Hz high-pass Butterworth filter (4^th^ order) and a 30 Hz low-pass Butterworth filter (9^th^ order) were applied to the continuous data. Ocular artifact correction was performed using the open-source BioSig package (BioSig, 2005) and an automated correction procedure (Schlögl et al., 2007). The continuous EEG data were then segmented 600 ms prior to motion stimulus onset and 9000 ms post-stimulus onset to capture both the Brownian noise and distraction under competition periods. Next, artifact correction and rejection procedures were applied to the segmented data using a robust procedure developed for data acquired through high density EEG systems. The Statistical Control of Artifacts in Dense Sensors Arrays (SCADS; Junghöfer et al., 2000) computes a compound data quality index for each participant at the individual channel and trial level. The compound data quality index is comprised of three statistical parameters with set thresholds including absolute mean amplitude, standard deviation, and maximum transient voltage change. Channel activity throughout the entire recording (global level) and at the single trial level was flagged for data quality when the quality index exceeded 2.5 standard deviations above the median index for that channel or trial, respectively. Flagged channels at the global and single trial level were interpolated by means of a 2D spline interpolation in which estimations of the values for the flagged channels were based on all channels. After interpolation, individual trials were flagged and rejected when the data quality index of that trial exceeded 1.25 times the median data quality index. After artifact rejection and correction, individual subject grand means were obtained by averaging surviving artifact free trials across the distractor picture conditions.

##### Postprocessing.

**Figure 3.**
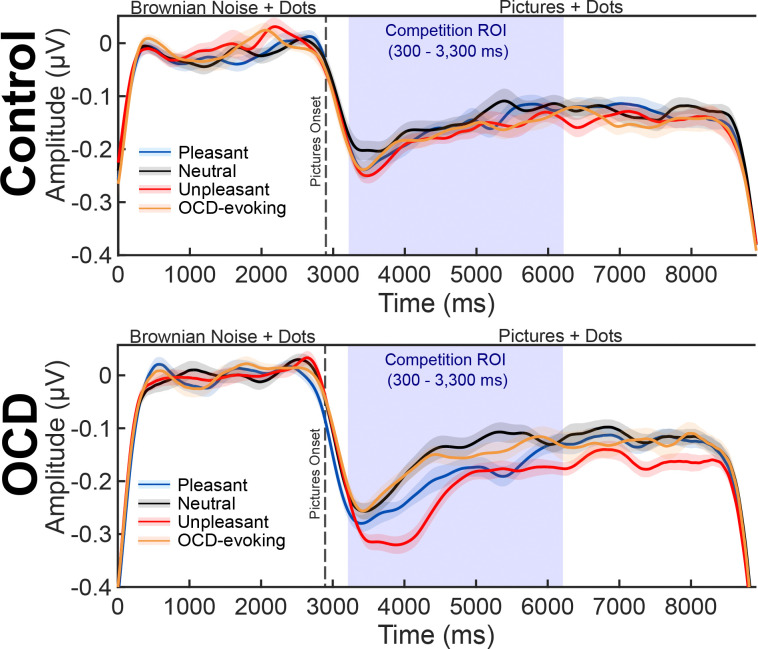
Evidence of decreased visuocortical engagement with task-relevant cues after distractor picture onset for both the control and OCD groups. Grand average ssVEP envelopes were computed across the sensors contributing to the interaction effect identified in the cluster-based permutation analysis. The shaded region around the solid lines indicates within-subject error.

The postprocessing pipeline functioned to transform the individual subject picture condition averages into time-varying estimates of the phase and amplitude of the steady-state evoked potential response to the flickering motion stimulus. To prepare for the transform, individual subject picture condition averages were referenced to the average reference and narrowly filtered using a band-pass Butterworth filter (9th order) with a lower frequency cut-off of 8.07 Hz and upper frequency cut-off of 9.07 Hz. The purpose of the narrow band-pass filter was to isolate the frequency band of interest, extracting the ssVEP response to the motion stimulus at 8.57 Hz. Next, the narrowly band-passed picture condition averages were Hilbert transformed using the FreqTag MATLAB toolbox (Figueira et al., 2022). Time-varying estimates of the amplitude and phase of the ssVEP response to the motion stimulus were extracted using the Hilbert transform to create an envelope of the signal across time. The resulting individual subject grand mean Hilbert envelopes provided continuous measures of visuocortical engagement with the task-relevant cues in visual cortex (Wieser et al., 2016). Individual subject grand mean responses were baseline adjusted to the average voltage of −2500 to −400 ms prior to picture onset and included in the mass univariate analysis approach. In addition, individual subject grand means for the OCD and control groups served as the task-evoked visual stream in the DUC model. The baseline adjustment time window extended throughout much of the Brownian noise period (excluding the first and last 400 ms to attenuate edge artifacts) to subtract the baseline ssVEP response to the motion stimulus in the absence of the competitive interaction (see Figure 3).

#### DUC model

##### Preprocessing.

**Figure 4.**
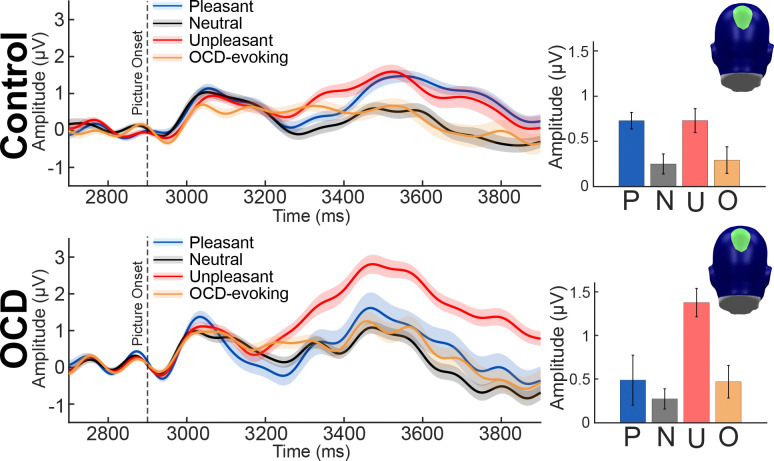
Picture-evoked time course included in the distraction under competition (DUC) model, and the average picture condition amplitudes across conditions (pleasant, P; neutral, N; unpleasant, U; OCD-evoking, O). Average picture-evoked amplitudes were computed over 1000 ms post-distractor onset and across the a priori parietal cluster of sensors shown in green on the topographical map. The shaded region around the solid lines indicates within-subject error.

The preprocessing pipeline for the picture-evoked responses used for DUC model fitting was virtually identical to the ssVEP preprocessing pipeline with the exception of the high- and low-pass filter parameters. The continuous EEG data were filtered using a 0.1 Hz high-pass Butterworth filter (4th order) and an 8 Hz low-pass Butterworth filter (10th order). The purpose of the strict low-pass filter was to extract the majority of the transient event related potential response from the oscillatory steady-state evoked potential response to obtain competing visual responses to the task-relevant motion stimulus and the affective distractor pictures, respectively across the same time course. This method has been applied in previous studies conducted by Müller & Hillyard (2000) and Schönwald & Müller (2014), demonstrating the feasibility of isolating transient stimulus-locked responses from steady-state signals using a low-pass filter with a lower cut-off frequency than the stimulation rate. Furthermore, the stimulation rate of 8.57 Hz guards against any potential overlap in the picture-evoked response and the task-evoked response (Bekhtereva et al., 2018). Individual subject grand means were obtained by averaging artifact free trials across the distractor picture content conditions. Individual subject grand means were baseline corrected to the average voltage 200 ms prior to distractor picture onset (see Figure 4).

##### Postprocessing.

Additional data preparation steps were taken to further isolate the distractor-evoked response from the task-evoked oscillatory signal and to standardize the two visual evoked response streams for optimal parametrization. The distractor-evoked signal was averaged across an *a priori* parietal-occipital cluster of 11 sensors surrounding electrode Pz (80, 89, 90, 100, 101 (Pz), 110, 118, 127, 128, 129, 130, 131), often included in analyses of sustained picture-evoked responses toward affective visual stimuli (Cuthbert et al., 2000; Hajcak et al., 2012; Keil et al., 2002; Schönwald & Müller, 2014; Weinberg & Hajcak, 2010). Similarly, the task-evoked response was averaged across sensor Oz. To account for the stimulus-locked nature of the picture-evoked signal, a duration of 1000 ms after distractor picture onset was included in the DUC model analysis. Both a 100-point centered moving average and 50 ms cosine window were applied to the individual subject distractor-evoked time courses across groups. Additional baseline correction was applied to the distractor-evoked time courses. The resulting task and distractor-evoked individual subject grand mean time courses were Z-transformed across groups and included in the DUC model.

### Statistical analyses

#### Mass univariate approach

##### Task-evoked responses

A mass univariate approach allowed for the examination of the ssVEP response without the need for rigid *a priori* sensor and time window determinations (Groppe et al., 2011). This approach is particularly well-suited for the statistical analysis of ssVEPs recorded using a high-density EEG system because of the increased spatial and temporal capabilities afforded with this technique (Groppe et al., 2011). The present analysis examined the emergence of competition effects across groups and conditions at each sensor and time point in the task-evoked response (Hilbert envelopes) to the motion stimulus after the distractor picture onset. We hypothesized that competition effects would differ across groups, and that these differences would be driven by the affective and OCD-evoking picture conditions. To this end, we completed mixed-factor planned contrasts with group-specific within-subject weights, placing greater weight on the affective and OCD-evoking picture conditions for the OCD group compared to the control group.

**Figure 5.**
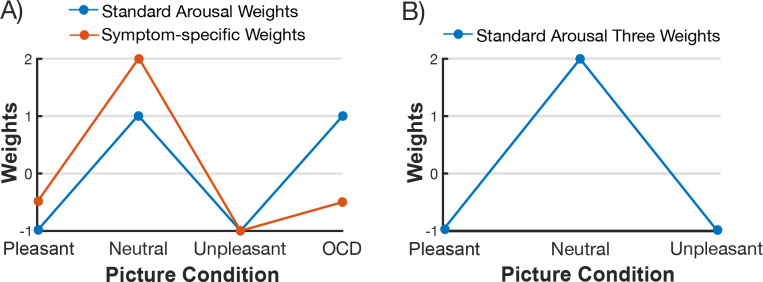
Graphical representation of the weights for the planned contrasts and control analysis. (A) For the mixed-factor planned contrast with within-subject specific weights the standard arousal weights were applied for the control group: −1 (Pleasant), 1 (Neutral), −1 (Unpleasant), 1 (OCD-evoking) and the for the OCD group the symptom-specific arousal weights were applied: −0.5 (Pleasant), 2 (Neutral), −1 (Unpleasant), −0.5 (OCD-evoking). (B) For the control analysis, the standard arousal three weights were applied: −1 (Pleasant), 2 (Neutral), −1 (Unpleasant).

The planned contrasts weights (see Figure 5A) for the control group were −1 (Pleasant), 1 (Neutral), −1 (Unpleasant), 1 (OCD-evoking) and the OCD group weights were −0.5 (Pleasant), 2 (Neutral), −1 (Unpleasant), −0.5 (OCD-evoking). As recommended by Rosenthal and Rosnow (1985), the error terms were aggregated across the picture content conditions. The denominator for each contrast (within, between, and interaction) was the within-subjects residual variance after subtracting subject and condition means. The error term was kept consistent across all contrasts because the error term aggregation procedure removed the mixed sources of variance between (i.e., subtracted the subject means) and within subjects (i.e., subtracted the condition means). The control group weights (standard arousal weights) were modeled after the characteristic, reliable pattern of electrocortical activation, often observed in nonclinical samples, where affective IAPS pictures captured greater attentional resources compared to neutral pictures (Bradley et al., 2012; Hindi Attar et al., 2010; Lang & Bradley, 2010; Müller et al., 2008; Schönwald & Müller, 2014). As we expected the responses to the OCD-evoking pictures to mirror the responses to neutral stimuli in the control group, the contrast weights were equivalent. Conversely, planned contrast weights for the OCD group were distributed so there was greater contrast magnitude, compared to the standard arousal weights, between the affective and OCD-evoking picture conditions and neutral picture condition (symptom-specific weights). Contrast weights differed between groups to examine overall group differences in competition driven by the affective and OCD-evoking picture conditions. Given the competing findings revealed in studies examining attention biases and disorder-relevant stimuli in OCD, equivalent weights were placed on the pleasant and OCD-evoking picture conditions.

To control for multiple comparisons, a nonparametric cluster-based permutation-controlled analysis was used to identify competition effects, across time and space (Maris & Oostenveld, 2007). The sensitivity of the cluster-based permutation analysis was optimized according to Maris and Oostenveld (2007): Biophysical constraints were placed on the sensors and time points included in the cluster-based permutation analysis. For instance, we expected to detect task-evoked ssVEP responses across parietal and occipital sensors, given that visual cortex primarily contributes to the emergence of ssVEP responses (Di Russo et al., 2006). Furthermore, studies employing variations of distraction under competition paradigms in anxious samples have shown reliable individual differences in competition persisting for several seconds after affective-picture onset (Deweese et al., 2014; Wieser et al., 2012). Therefore, occipital-parietal sensors and time-points spanning a 3 second *a priori* competition window of interest beginning 300 ms after picture onset were included in the permutation analysis. Statistically significant clusters were identified via a conservative F_max_ procedure, increasing our ability to reliably detect large effects (Tebbe et al., 2026). Permuted within-subjects, between-subjects, and interaction F-scores were calculated separately across 1,000 permutations at each sensor and time point included in the analysis. Subjects included in each of the permutations were randomly sampled with replacement and their corresponding picture condition labels were shuffled. Mass univariate and cluster-based permutation analyses were carried out using custom Matlab scripts available in the project repository (https://osf.io/s73d5/overview).

##### Control analysis

In addition, a control analysis was carried out to evaluate visuocortical engagement with task-cues in response to the affective and neutral picture conditions, excluding the OCD-evoking picture condition from the planned contrast (see Figure 5B; three weight contrast: −1 (Pleasant), 2 (Neutral), −1 (Unpleasant)). The control analysis was constrained by the same cluster-based permutation-controlled analysis detailed above.

### Modeling visuocortical trade-off driven by attention biases

The DUC model was first presented as an extension of the normalization model of attention (Reynolds & Heeger, 2009) to expand the model’s ability to characterize local field potential responses, such as ssVEPs, rather than individual neuronal activity toward competing stimuli (De Echegaray et al., 2024; Müller et al., 2026). Furthermore, the DUC model utilizes time-delayed model infrastructure to overcome the time-invariant constraints of the normalization model of attention, and dynamically model competitive interactions occurring over hundreds of milliseconds (De Echegaray et al., 2024). The DUC model includes three parameters of interest to model the temporal dynamics of visual evoked potentials. The β_D_ parameter models the initial content-selective response to the distractor stimulus, the ϕ parameter models the early inhibitory influence of the distractor cues on the task-relevant cues, and the λ parameter models sustained competition between the distractor and task-evoked response (De Echegaray et al., 2024; Müller et al., 2026).

In an identical procedure to De Echegaray et al. (2024) and Müller et al. (2026), the DUC model was fit to the empirical data using non-linear regression (nlinfit in MATLAB) on 5,000 bootstrapped grand mean task-evoked envelopes and picture-evoked waveforms. Grand average task-evoked envelopes and picture-evoked waveforms were computed by resampling participants with replacement. Grand average envelope and waveform magnitude was expressed as percent change from baseline, and all model parameters varied freely. Finally, a non-parametric Bayesian bootstrap approach (Ahumada et al., 2025; Efron, 2011) was used to compare the distributions of the parameter estimates across picture conditions and groups. Mean square error (MSE) was calculated across each distribution and the same Bayesian bootstrap approach was used to compare model fit indices within and across groups. The present application of the DUC model was performed using custom Matlab scripts available in the project repository (https://osf.io/s73d5/overview).

### Behavioral data

Task performance was also analyzed using the same mixed-factor planned contrasts with the group-specific within-subject weights detailed above to examine the effects of picture content (pleasant, neutral, unpleasant, and OCD-evoking) and group (OCD and control) on task accuracy. Task accuracy was quantified as the proportion of correct responses (indicated ‘yes’ when there was a coherent motion event in target trials and ‘no’ when there was no coherent motion on non-target trials) to the total number of responses.

## Results

### Demographics

Demographic data were examined across groups (see Table 1) and indicated that the control and OCD groups did not differ across gender (*X*^2^(2, *N* = 64) = 2.59, p = 0.27, Cramer’s V = 0.20), race (*X*^2^(4, *N* = 64) = 7.41, p = 0.12, Cramer’s V = 0.34), or ethnicity *X*^2^(1, *N* = 64) = 0.27, p = 0.60, Cramer’s V = 0.07. However, participants in the control group (M = 38.13, SD = 18.97) were significantly older (t(43.16) = 2.84, p < .05, *d* = 0.26) compared to the participants in the OCD group (M = 27.42, SD = 9.35 years).

### Trial counts

Repeated measures ANOVAs were carried out to examine the number of trials included in the analyses. There were no significant differences in the number of trials rejected across conditions after application of the ssVEP preprocessing pipeline (F(3, 186) = 0.43, p = 0.73, partial η^2^ = 0.01). However, there was a significant difference in the number of trials rejected due to poor data quality between groups (F(1, 62) = 4.42, p = 0.04, partial η^2^ = 0.07). Descriptively, the average number of ssVEP trials rejected in the control group was about one trial more than the average number of trials rejected in the OCD group, regardless of condition. For the picture-evoked preprocessing pipeline, there were no significant differences in the number of trials rejected between groups (F(1, 62) = 1.29, p = 0.26, partial η^2^ = 0.02) or across conditions (F(3, 186) = 0.70, p = 0.55, partial η^2^ = 0.01). Similarly, the average number of picture-evoked trials rejected in the control group was just under one trial more than the average number of trials rejected in the OCD group, regardless of condition.

### Behavioral data

**Figure 6.**
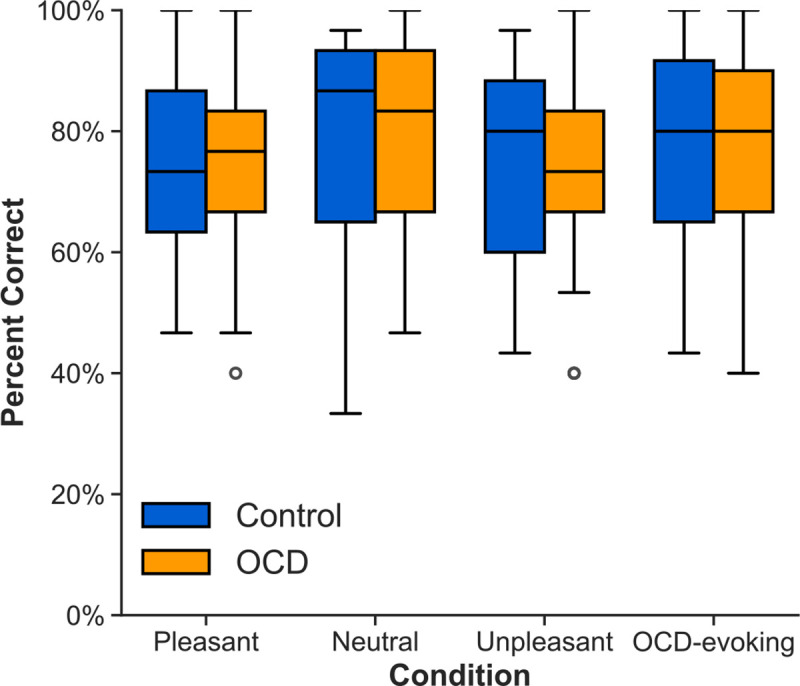
Average task performance across conditions and groups. Task accuracy was quantified as the proportion of correct responses (indicated ‘yes’ when there was a coherent motion event in target trials and ‘no’ when there was no coherent motion on non-target trials) to the total number of responses.

The results of the mixed-factor planned contrasts revealed a significant effect of condition (F(1, 192) = 15.16, p < .001, partial η^2^ = 0.02) on task accuracy. However, the effect of group was not significant (F(1, 192) = 0.02, p = 0.88, partial η^2^ = 0.0001). Regardless of group, these results indicated poorer task performance on trials where affective and disorder-relevant pictures were presented compared to neutral pictures (see Figure 6).

### Mass univariate analysis across time and sensors

**Figure 7.**
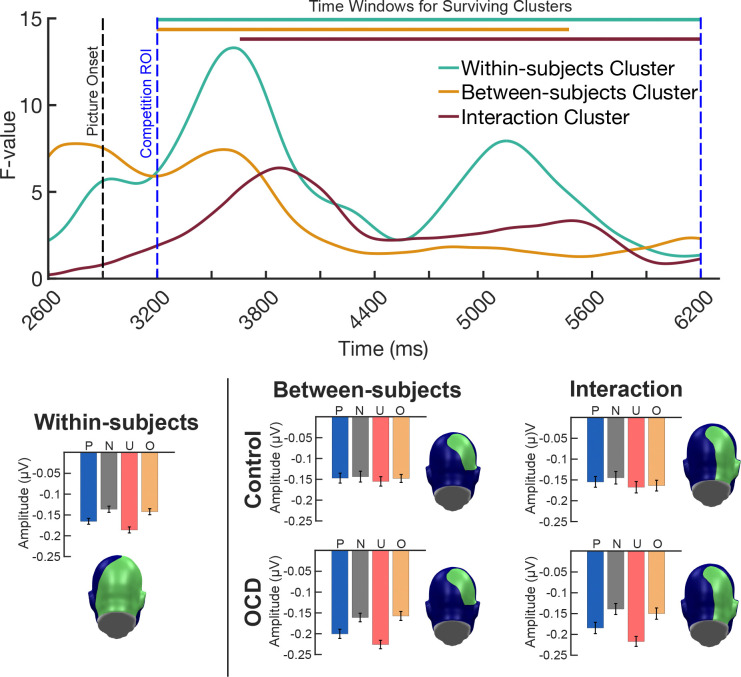
Evidence of group differences exhibiting stronger competition effects for the OCD group, driven largely by the unpleasant pictures, and less so by the OCD-evoking pictures. Time courses were obtained by averaging the resulting F-values from the mass univariate mixed-factor planned contrasts, across the sensors identified in each of the clusters that survived cluster-based permutation analysis. Colored bars at the top of the time course indicated the length of the time window identified for each cluster that survived cluster-based permutation analysis. Topographies indicate the sensors identified in the within-subjects, between-subjects, and interaction clusters that survived cluster-based permutation analysis. Average task-evoked envelope amplitudes for each condition conditions (pleasant, P; neutral, N; unpleasant, U; OCD-evoking, O) were computed across the within-subjects, between-subjects, and interaction cluster time window and sensors are shown as bar plots.

Cluster-based permutation analysis revealed statistically significant clusters of sensors across time exhibiting cluster sums (F-value sum for each cluster) greater than the permuted F-maximum (maximum cluster sum of permuted F-values) for the within-subjects, between-subjects, and interaction mixed-factor planned contrasts (see Figure 7). Within subjects, a large cluster containing 101 occipital-parietal sensors over medial posterior regions exceeded (∑F-value_observed_ = 710,269) the permuted within-subjects F-maximum (∑F-value_perm_ = 26,950) and spanned the entire *a priori* competition time window of interest (3,200 ms to 6,200 ms or 300 ms to 3,300 ms post picture onset). Between subjects, a moderately sized cluster emerged across 27 right lateralized occipital-parietal sensors exhibited a cluster sum (∑F-value_observed_ = 97,221) exceeding the permuted between-subjects F-maximum (∑F-value_perm_ = 14,732) and spanned a considerable amount of the competition time window from 3,200 ms to 5,574 ms. The interaction cluster included 45 right lateralized occipital-parietal sensors and displayed a cluster sum (∑F-value_observed_ = 119,798) surpassing the permuted interaction F-maximum (∑F-value_perm_ = 93,685). Significant individual differences in competition were detected around 760 ms after picture onset and persisted across the remainder of the *a priori* competition time window (3,660 ms - 6,200).

### Control analysis

In addition, the control analysis revealed a significant main effect of condition, but not of group when the OCD-evoking picture condition was left out of the analysis. The resulting cluster included 97 occipital-parietal sensors, which were subsumed in the within-subjects cluster previously identified in the 8-weight cluster-based permutation analysis. The cluster sum (∑F-value_observed_ = 533,011) exceeded the permuted F-maximum (∑F-value_perm_ = 7,781) and again spanned the entire *a priori* competition time window interest (3,300 ms to 6,300 ms or 300 ms to 3,300 ms post-picture onset).

### DUC Model Fitting

Table 2 shows the best-fitting parameter estimates by condition for each of the three parameters of interest. Also included in Table 2 are the resulting log base 10 Bayes factors (Log10BF) computed using a Bayesian bootstrap approach (Ahumada et al., 2025; Efron, 2011) to compare the bootstrapped distributions of the parameter estimates across conditions. Bayes factors were interpreted according to Jeffreys (1961), where log10BF values greater than |1| were considered strong evidence, values greater than |1.5| were considered very strong evidence, and values greater than |2| were considered decisive evidence of an effect. Bayesian comparisons across picture conditions resulted in an overall trend of increased affective picture interference (enhanced neural responses toward emotional stimuli compared to neutral stimuli; Bradley et al., 2012) in the pleasant and unpleasant picture conditions for the control group, and the unpleasant picture condition for the OCD group. For the OCD group, the content-selective parameter comparison across the unpleasant and neutral picture conditions revealed decisive evidence of an increased initial content-selective response toward the unpleasant pictures. For the control group, the content-selective parameter comparison indicated strong to very strong evidence of an increased initial content-selective response for the unpleasant and pleasant pictures, respectively. Table 3 shows the Bayes factors (Log10BF) obtained for each comparison of the parameter estimates across groups. Overall, comparisons demonstrated the magnitude of the visuocortical competition in response to the unpleasant pictures in the OCD group compared to the control group. Furthermore, comparisons displayed substantial evidence of increased content-selective, early distractor inhibitory influence on task responses.

**Figure 8.**
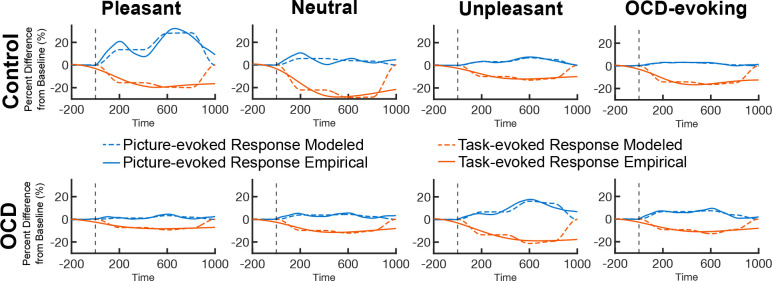
Best-fitting picture-evoked and task-evoked modeled responses fit to empirical picture-evoked and task-evoked responses.

The DUC model exhibited good fit with the empirical data (see Figure 8). Model fit within groups was assessed using average mean square error (mMSE) computed across the distributions of the MSE values obtained for each resample (see Table 4). Model fit across groups was examined using the same Bayesian bootstrap approach where distributions of MSE values for each condition were compared across groups. Positive log10BF values indicated greater model fit for the OCD group and negative log10BF values indicated greater model fit for the control group. Bayes comparisons showed strong to very strong evidence of greater model fit in the OCD group for the neutral (log10BF = 1.41) and pleasant (log10BF = 1.95) picture conditions, respectively. Comparisons showed similarly strong evidence of greater model fit in the control group for the unpleasant (log10BF = −1.32) picture condition. For the OCD-evoking condition, Bayes comparisons showed anecdotal evidence of greater model fit in the OCD group for the OCD-evoking (log10BF = .44) picture condition.

## Discussion

The current study examined the extent to which task and task-irrelevant affective and disorder-relevant cues competed for limited capacity attention in participants with OCD and controls. Competition in visual cortex was continuously measured using ssVEPs to quantify the visuocortical trade-off between affective and disorder-relevant distractor pictures and neutral task cues. Furthermore, the DUC model, a framework for selective attention, was applied to the data to obtain meaningful parameter estimates of the competitive interactions between the distractor pictures and task-relevant cues in visual cortex (De Echegaray et al., 2024; Müller et al., 2026).

Mass univariate results indicated the task-evoked ssVEP envelopes in both groups were attenuated upon presentation of the distractor pictures, more so for affective compared to neutral pictures. Regardless of group, the data showed increased visuocortical competition effects in response to the affective pictures (pleasant and unpleasant) across a substantial number of medial occipital-parietal sensors over the entire *a priori* competition window (300 ms to 3,300 ms post-picture onset). In addition, individual differences were observed over a large portion of right lateralized occipital-parietal sensors and began around 760 ms after affective picture onset and persisted throughout the *a priori* competition window. The lateralization of the interaction effect is not surprising in the light of past research: Functional magnetic resonance imaging (fMRI) has shown increased bilateral BOLD activation in the occipital gyrus, and right lateralized BOLD activation in the inferior and superior parietal lobes in response to emotional, compared to neutral pictures (Lang et al., 1998). In addition, the time course of our results aligned with both the onset and duration of the group differences in competition identified by Wieser and colleagues (2012) and Deweese and colleagues (2014) in anxious samples. Furthermore, the interaction effect indicated increased visuocortical competition in the OCD group compared to the control group, driven primarily by the attention bias to unpleasant pictures, followed by pleasant and OCD-evoking pictures.

Consistent with our first hypothesis, affective distractor content impacted visuocortical engagement with task cues, driven primarily by the unpleasant and pleasant picture conditions, regardless of group. The emotional impact of picture condition on task-evoked ssVEP envelope amplitudes align with findings in several previous studies employing distraction under competition paradigms (De Echegaray et al., 2024; Deweese et al., 2014, 2016; Hindi Attar et al., 2010; Müller et al., 2008; Schönwald & Müller, 2014). Accumulating evidence has demonstrated visuocortical facilitation toward emotional pictures (pleasant and unpleasant IAPS pictures), often at the cost of concurrently presented neutral task-relevant cues (Bradley et al., 2012; Deweese et al., 2016; Hindi Attar et al., 2010; Keil et al., 2005). Moreover, group differences in overall visuocortical trade-off can be explained by patterns of altered visual attention processing along the visual attention stream in participants with OCD. Evidence of altered early visual processing in OCD has emerged in several EEG investigations examining event-related visual evoked potential (ERPs) responses (for a review, see Ayomen et al., 2025). For instance, individuals with OCD showed enhanced P1 (Chapman et al., 2023) and N2 (Lei et al., 2015; Riesel et al., 2017) amplitudes in response to neutral cues in paradigms aimed to capture evidence of altered early visual processing and response inhibition, respectively. In addition to broadly altered early visual perception in individuals with OCD, an attentional bias toward aversive or threat-related visual information tends to emerge across psychophysiological investigations in individuals with OCD (for a review, see Cooper & Dunsmoor, 2021).

Moreover, fear conditioning paradigms have been used to examine attention biases in individuals with OCD and tend to provide evidence of altered fear extinction and overgeneralized threat responses (Apergis-Schoute et al., 2017; Cooper & Dunsmoor, 2021; Elsner et al., 2022; Milad et al., 2013).

The interaction effect described above provides evidence of altered attention implementation in individuals with OCD, revealing increased competitive visuocortical interactions between task and task-irrelevant pictures as a function of picture content. Attention biases emerged across both groups and indicated stronger competition effects in the OCD group, driven to a large extent by the unpleasant pictures, followed by the pleasant and OCD-evoking pictures. However, the magnitude of the visuocortical competition observed in the unpleasant and pleasant picture conditions was not readily observed in the OCD-evoking picture condition. Furthermore, the subjective valence and arousal ratings also show considerable differences between the unpleasant and OCD-evoking picture conditions. Previous research examining specific picture categories within the unpleasant IAPS pictures may offer insight into the substantial competition observed in the unpleasant picture condition for the OCD group. Several fMRI studies examining BOLD activation in response to subsets of the unpleasant IAPS pictures in participants with checking, symmetry, harm, and predominantly contamination obsessions revealed unique patterns activation in response to both disgust-inducing and threat-related pictures taken from the IAPS (Berlin et al., 2015; Shapira et al., 2003; Stark et al., 2003). In these studies, the disgust-inducing pictures were chosen from the unpleasant IAPS picture category and overlapped with the unpleasant pictures used in the present study. Considering the impact of disgust-inducing pictures in participants with OCD, the present unpleasant picture condition may have served as a disorder-relevant picture category. The present study falls under a large-scale research initiative investigating obsessive-compulsive anxiety spectrum disorders, and individual picture level analyses will be conducted once the final sample is collected to examine individual visuocortical competition in response to specific picture subtypes.

Interestingly, the control analysis indicated attenuated group differences in the magnitude of the visuocortical competition in response to the pleasant and unpleasant distractor pictures when the OCD-evoking picture condition was excluded from the planned contrasts. The results of the control and behavioral analysis may reflect the tendency for paradigms presenting pleasant, neutral, and unpleasant IAPS pictures to reliably produce robust visuocortical and behavioral indices of emotional processing (Cuthbert et al., 2000; Keil et al., 2002). In addition, the results of the SAM evaluation ratings suggest consistency across individual valence ratings for the pleasant, neutral, and unpleasant picture conditions, regardless of group. However, measuring individual differences from robust, reliable indices of cognition are thought to be a contributing factor in the “reliability paradox” proposed by Hedge and colleagues (2018). The authors provide evidence supporting the notion that robust cognitive and behavioral tasks may not be suitable for the examination of individual differences due to high reliability of responses elicited in these tasks. To combat the reliability paradox, Haines and colleagues (2025) advocate for use of generative models of cognitive constructs to obtain meaningful parameter estimates of cognitive processes. Therefore, the present investigation applied the DUC model to quantify the visuocortical trade-off between the task-evoked and picture-evoked responses for each picture condition, and across groups (De Echegaray et al., 2024; Müller et al., 2026).

Modeling competitive interactions in visual cortex revealed good model fit across groups, supporting our hypothesis and corroborating a shift of visuocortical engagement with task-relevant cues to the distractor picture. DUC model comparisons of the content-selective parameter distributions across groups seemed to suggest unpleasant pictures evoked the largest initial picture content-driven response in the OCD group. In contrast, for the control group the pleasant pictures evoked substantial initial responses to picture content. Overall, comparisons across groups indicated more competition in response to the unpleasant and disorder-relevant picture conditions in the OCD group compared to the control group. Furthermore, comparisons across groups confirmed the strength of the attentional impact of the unpleasant pictures in the OCD group, supporting the results of the mass univariate analysis. However, comparisons also indicated that the OCD-evoking pictures substantially impacted attentional engagement with the task-relevant cues in the OCD group compared to the control group. The visuocortical impact of the OCD-evoking pictures on the OCD group was not readily observed in the univariate analysis.

## Conclusion

Establishing objective, dimensional indices of mental health is at the forefront of recent efforts to bridge physiological and clinical research efforts through integrated frameworks of psychopathology (Cuthbert, 2014; Lake et al., 2017; Patrick & Hajcak, 2016). Visual processing occupies the cognitive processing domain of one such framework: the National Institutes of Mental Health Research Domain Criteria (RDoC) proposed by Insel and colleagues Insel et al. (2010). Consequently, research on individual differences has gained traction in the field of electrophysiology, reflected in numerous investigations aimed to evaluate altered visual processing in individuals along the obsessive-compulsive anxiety spectrum of disorders (for a review, see Ayomen et al., 2025; Figueira et al., 2024; Riesel, 2019; Riesel et al., 2017). However, speculation about the feasibility of integrating psychophysiological and behavioral indices of mental health with current diagnostic clinical indices remains rooted in reliability concerns (Weinberger et al., 2015). In response, the present study followed recommendations proposed by Haines et al. (2025) to bridge the so-called “theory-description gap”, ensuring our outcome measures and analyses are bound by theoretically informed constraints. Therefore, participants with OCD exhibited altered visual attention implementation under competition, driven by an attentional bias toward unpleasant distraction, followed by disorder-relevant distraction.

## Figures and Tables

**Table 1.** Demographic data presented as frequency and percentage.

		Total (n = 64)	Control Group (n = 31)		OCD Group (n = 33)	

		Frequency	Frequency	Percentage	Frequency	Percentage

Gender^a^	Female	49	22	71.0	27	81.8
	Male	14	9	29.0	5	15.2
	Prefer not to answer	1	0	0.0	1	3.0

Race^b^	Asian	3	3	9.7	0	0.0
	American Indian/Alaskan Native	1	0	0.0	1	3.0
	Black/African American	5	4	12.9	1	3.0
	Unknown/Prefer not to answer	1	0	0.0	1	3.0
	White/Caucasian	54	24	77.4	30	91

Ethnicity^c^	Hispanic/Latino	12	5	16.1	7	21.2
	Non-Hispanic/Non-Latino	52	26	83.9	26	78.8

Age^d^	<19	5	2	6.5	3	9.1
	20–29	34	13	41.9	21	63.7
	30–39	9	5	16.1	4	12.1
	40–49	4	0	0.0	4	12.1
	50–59	6	5	16.1	1	3.0
	>60	6	6	19.4	0	0.0
	Average and standard deviation		38.1(19.0)		27.4(9.4)	

a Chi-square test showed no significant differences in gender across groups *X*^2^(2, *N* = 64) = 2.59, p = 0.27, Cramer’s V = 0.20.

b Chi-square test showed no significant differences in race across groups *X*^2^(4, *N* = 64) = 7.41, p = 0.12, Cramer’s V = 0.34.

c Chi-square test showed no significant differences in ethnicity across groups *X*^2^(1, *N* = 64) = 0.27, p = 0.60, Cramer’s V = 0.07.

d Independent Welch’s t-test showed significant differences in age across groups t(43.16) = 2.84, p < .05, *d* = 0.26.

**Table 2.** Best fitting DUC model parameter estimates of the three parameters of interest can be seen in the first four columns.

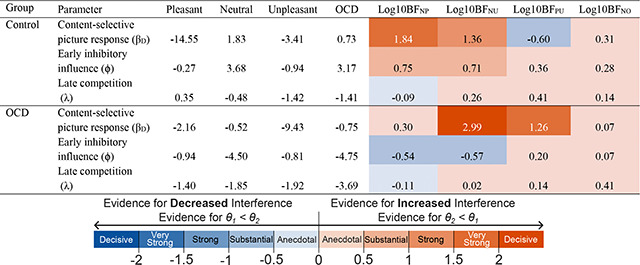

In addition, comparisons of the parameter estimate distributions by means of a Bayesian bootstrap procedure can be seen in the last four columns. Bootstrap distributions were compared within groups: neutral (*θ_1_*, N) compared to pleasant (*θ_2_*, P), neutral (*θ_1_*, N) compared to unpleasant (*θ_2_*, U), pleasant (*θ_1_*, P) compared to unpleasant (*θ_2_*, U), and neutral (*θ_1_*, N) compared to OCD-evoking (*θ_2_*, O) picture conditions. For the comparisons between affective and neutral picture conditions, greater content-selective initial response (β_D_), early distractor inhibitory influence (ϕ), or late competition (λ) in response to affective and disorder-relevant pictures (increased interference) was indicated by positive log base 10 Bayes factors (log10BF) and negative log10BF values indicated increased β_D_, ϕ, or λ in response to the neutral pictures (decreased interference). For the pleasant (*θ_1_*, P) and unpleasant (*θ_2_*, U) picture condition comparison, positive log10BF values indicated increased β_D_, ϕ, or λ in response to the unpleasant pictures, and negative log10BF values indicated increased β_D_, ϕ, or λ in response to the pleasant pictures. log10BF values were interpreted according to Jeffreys (1961) scale where values greater than 1 indicated strong support for an effect.

**Table 3.** Group comparison of parameter estimate bootstrap distributions using the Bayesian bootstrap procedure (Log10BF) and interpretation reported in Table 2.

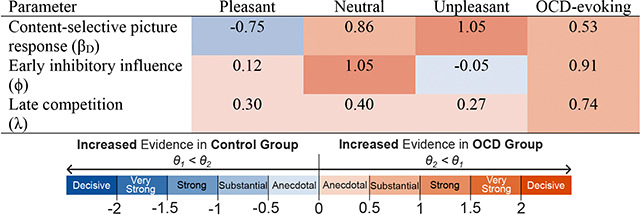

Bootstrap distributions of the control group (*θ_1_*) were compared to the OCD group (*θ_2_*). Positive log base 10 Bayes factors (log10BF) indicated increased content-selective initial response (β_D_), early distractor inhibitory influence (ϕ), or late competition (λ) in the OCD group. Negative log base 10 Bayes factors (log10BF) indicated increased content-selective initial response (β_D_), early distractor inhibitory influence (ϕ), or late competition (λ) in the OCD group.

**Table 4.** DUC model fit within groups. Average mean square error (mMSE) values were computed across distributions of the MSE values obtained for each of the 5,000 draws.

	Condition	mMSE

**Control**	Pleasant	11.14
	Neutral	15.52
	Unpleasant	3.35
	OCD-evoking	4.36

**OCD**	Pleasant	2.29
	Neutral	2.66
	Unpleasant	8.89
	OCD-evoking	2.84
