# Supplementary material for: Heightened Distraction under Competition in Obsessive-Compulsive Disorder
Source: bioRxiv. 2026 May 21:2026.03.15.711932. Originally published 2026 Mar 17. Preprint. [Version 2] doi: 10.64898/2026.03.15.711932 (PMC13015311; doi:10.64898/2026.03.15.711932)
Supplement: Supplement 1 [file media-1.pdf]

Supplemental Material

Supplemental Table 1. Demographic data presented as frequency and percentage.

		Total (n = 64)	Control Group (n = 31)		OCD Group (n = 33)	
		Frequency	Frequency	Percentage	Frequency	Percentage
Gender[a]	Female	49	22	71.0	27	81.8
	Male	14	9	29.0	5	15.2
	Prefer not to answer	1	0	0.0	1	3.0
Race[b]	Asian	3	3	9.7	0	0.0
	American Indian/Alaskan Native	1	0	0.0	1	3.0
	Black/African American	5	4	12.9	1	3.0
	Unknown/Prefer not to answer	1	0	0.0	1	3.0
	White/ Caucasian	54	24	77.4	30	91
Ethnicity[c]	Hispanic/Latino	12	5	16.1	7	21.2
	Non-Hispanic/Non-Latino	52	26	83.9	26	78.8
Age[d]	<19	5	2	6.5	3	9.1
	20-29	34	13	41.9	21	63.7
	30-39	9	5	16.1	4	12.1
	40-49	4	0	0.0	4	12.1
	50-59	6	5	16.1	1	3.0
	>60	6	6	19.4	0	0.0
	Average and standard deviation		38.1(19.0)		27.4(9.4)	

[a]Chi-square test showed no significant differences in gender across groups $X^2(2, N = 64) = 2.59$, p = 0.27, Cramer's V = 0.20.
[b]Chi-square test showed no significant differences in race across groups $X^2(4, N = 64) = 7.41$, p = 0.12, Cramer's V = 0.34.
[c]Chi-square test showed no significant differences in ethnicity across groups $X^2(1, N = 64) = 0.27$, p = 0.60, Cramer's V = 0.07.
[d]Independent Welch's t-test showed significant differences in age across groups $t(43.16) = 2.84$, p = .007, d = 0.72.
